# Importance of the Matrix and the Matrix/Sample Ratio in MALDI-TOF-MS Analysis of Cathelicidins Obtained from Porcine Neutrophils

**DOI:** 10.1007/s12010-014-1405-1

**Published:** 2014-11-29

**Authors:** Anna Smolira, Joanna Wessely-Szponder

**Affiliations:** 1 Department of Molecular Physics, Institute of Physics, Maria Curie Sklodowska University, Pl. M. Curie-Skłodowskiej 1, 20-031 Lublin, Poland; 2 Department of Pathophysiology, Chair of Preclinical Veterinary Sciences, Faculty of Veterinary Medicine, University of Life Sciences, Akademicka 12, 20-033 Lublin, Poland

**Keywords:** MALDI TOF MS, Sensitivity, Sample preparation, Matrix, Matrix/sample ratio, Cathelicidins, Antimicrobial peptides

## Abstract

Qualitative and quantitative mass spectrometric studies of biomolecules for example proteins, peptides, or lipids contained in biological samples like physiologic fluids are very important for many fields of science such as medicine, veterinary medicine, biology, biochemistry, molecular biology, or environmental sciences. In the last two decades, MALDI TOF MS — matrix-assisted laser desorption mass spectrometry, proved to be an especially convenient tool for these analyses. The main advantages of this method are its rapidity and high sensitivity which is particularly appreciated in the case of studies of complex biological specimen. A major challenge for many researchers is to maximize this sensitivity, among others, by appropriate procedures of sample preparation for the measurement. The objective of this work was to optimize these procedures, selecting the optimal matrix and optimum proportions of the sample and the matrix solution in a mixture of both solutions, aiming at the achievement of the maximum intensity of ion current. In this respect, five low molecular mass cathelicidins were studied: prophenin-2, protegrins 1–3, PR-39. All of them were obtained directly from the porcine blood. As a result of studies, the authors determined such experimental conditions when the intensity of investigated ionic current had the highest value.

## Introduction

The MALDI method (matrix-assisted laser desorption ionization) in combination with a time of flight mass spectrometer (TOF MS) is an indispensable tool for the detection and identification of molecular masses of “heavy” biomolecules, which due to thermal instability can not be mass analyzed by the laser desorption (LD) method that is traditionally used to measure small masses [1, 2]. MALDI-TOF MS is also very helpful in mass spectrometric studies of low molecular mass biomolecules [3]. For example, peptides smaller than 7000 Da are still too “heavy” for the LD TOF MS measurement and simultaneously can not be detected by ordinary techniques of two-dimensional electrophoresis because they are below the limits of size resolution and small components may not be fixed in gel and produce lower staining intensity per mole of peptide. Most electrophoretic and chromatographic techniques preferentially detect large molecules because absorbance or intensity of staining per molar equivalent of protein generally increases in proportion to size. Consequently, MALDI-TOF MS has served as a useful tool for identification and characterization of small and heavy peptides as well as for peptidomic analysis [4–6].

MALDI TOF MS is the method that provides rapid determination of molecular masses. Its attribute is also high sensitivity and no need for prior purification of the samples before the measurement, which is especially important in studies of biological samples with for example proteins, oligosaccharides, lipids, or peptides contained in them [6–15]. However, in the case of samples with large amounts of buffers, they should be desalted prior to a measurement [16, 17]. Detection limits depend on many variables that may change detection sensitivity. For small peptides, under optimal conditions, detection limits can extend to 1 nmol/l solution in 1 μl.

One of the factors that influences MALDI TOF MS analyses both in terms of sensitivity and resolution is sample preparation. Thus, the major challenge for such studies is to identify its optimal procedures. This is of particular importance in the case of biological specimen contained in physiologic fluids where very often low amounts of an investigated material are available for experiment, and additionally, it is complex and not purified. Since the introduction of MALDI in 1988, different procedures in the subsequent stages of sample preparation were tested:

- preconcentration of the sample — for example coating of the sample holder surface with hydrophobic or omniphobic materials such as 3M Scotchgard [18], paraffin wax film [19, 20], polytetrafluoroethylene [21, 22], mineral oil, glycerol, vaseline [23], hydrophobic foil [24]; placing the sample into nanovials [25–28] or onto hydrophilic areas of hydrophobic material [29],
- purification (desalting) of the sample — for this purpose, there can be used for example films of commercial polymers, thin layers of matrix crystals or ultrathin polymer films [30], drop dialysis [31], and self-assembled monolayer (SAM) technique [32–34],
- using a suitable matrix [35–38] or micro- and nanostructured targets that could serve as a matrix for example: the DIOS method (desorption and ionization on porous silicon) developed in 1999 by Siuzdak and coworkers [39], nanostructure initiator mass spectrometry (NIMS) [40], surface-enhanced laser desorption ionization (SELDI) [41], or self-assembled monolayers desorption ionization (SAMDI) [42],
- preparation of the sample and the matrix solutions based on appropriate solvents (solvent-based MALDI MS) [43] or alternatively using solvent-free sample preparation method [44],
- in the case of solvent-based MALDI MS, mixing these two solutions in the right proportions [44, 45],
- using the most appropriate sample deposition method (dried droplet [46–49], thin- [50] or seed-layered [51], spin-coated [52], electrospray [53–55], “acetone redeposition” [56], and aerospray [57–59]) in order to get good homogeneity of the crystallized sample/matrix mixture and high degree of shot-to-shot, spot-to-spot, and sample-to-sample reproducibility of the obtained results.

The aim of the presented paper is to show the relationship between the matrix/sample ratio used in the MALDI sample preparation process and the quality of the obtained mass spectra. The literature concerning this topic refers mainly to commercially available samples that are composed of one type of an analyte such as for example cytochrome c [60], bovine albumin [60], or polymethylmethacrylate (PMMA) [44]. These reports indicate clearly that the matrix/sample ratio is a critical parameter when using solvent-based MALDI MS [44].

The MALDI TOF MS method was previously used by the authors for determination of the content of neutrophil extract [61, 66, 67]. The cathelicidins contained in it were identified according to their molecular masses. Their activity was confirmed by antimicrobial tests against E. coli — minimal inhibitory concentrations (MIC) and bactericidal activity (MBC) [61].

The presented investigations were carried out using samples (portions) of lyophilisate from porcine neutrophils (see “Materials and Methods”) consisting of, inter alia, low molecular mass cathelicidins: proline-phenylalanine-rich prophenin-2 (PF-2, *M* = 8807 Da), proline-arginine-rich 39-amino-acid peptide (PR-39, *M* = 4716 Da), cysteine-rich protegrins 1–3 (PG-1, *M* = 2154.5 Da; PG-2, *M* = 1955.6 Da; PG-3, *M* = 2055.5 Da).

PF-2, PR-39, PG-1, PG-2, and PG-3 belong to antimicrobial peptides with a direct antimicrobial effect, as well as a wide range of activities on the immune system, which can be taken into account when introducing new antibiotic treatments [61]. They are of particular interest for many research scientists in the field of veterinary medicine where an accurate identification of their masses is often essential for any subsequent investigations.

The studies were devoted to the problem of optimization of the sample preparation process for the MALDI TOF MS measurement to get sensitivity of the investigated cathelicidin detection as high as it was possible. In particular, the authors focused on selecting the optimal matrix and determination such ratio of a matrix and a sample solution (*v*/*v*) in the measured mixture of both solutions when the intensity of ionic current of each studied cathelicidin had the highest value.

## Experimental

### MALDI-TOF Instrumentation

**Fig. 1:**
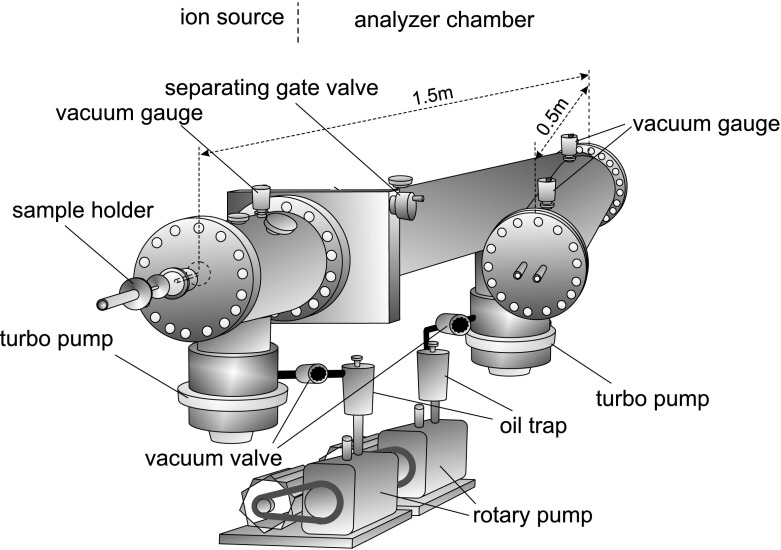
The scheme of the time of flight mass spectrometer used in the presented investigations. The apparatus was built by the author and coworkers in the Department of Molecular Physics, Institute of Physics, Maria Curie Sklodowska University

**Fig. 2:**
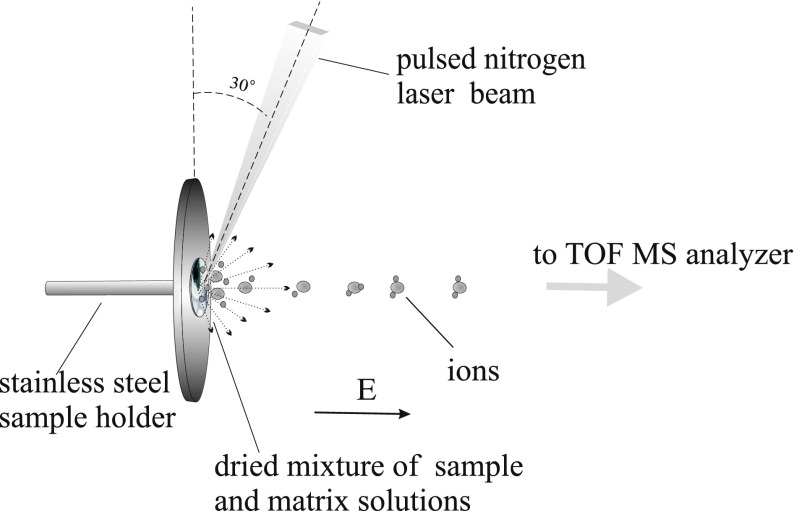
The MALDI TOF MS sample holder with the matrix and the sample on its surface. The analyte ions created in the ion source near the sample holder surface are directed to the TOF mass analyzer

The MALDI TOF MS analysis was performed on the time of flight mass spectrometer constructed by the author and co-workers in the Department of Molecular Physics, Institute of Physics, Maria Curie—Sklodowska University, Lublin, Poland (Fig. 1). In the presented studies, the linear mode of the spectrometer was used. Detailed parameters of the apparatus have already been discussed elsewhere [62]. The molecules are ionized with the nitrogen laser (LNC300C; Laser Photonics, New York, NY, USA, *λ* = 337 nm, an output length of 5 ns, a maximum pulse energy specified as 250 μJ, and a rectangular shape of approximately 4 × 9 mm). The laser beam is focused using a quartz lens (*f* = 15 mm) at an angle of 30° to the surface of the sample holder (Fig. 2). The power density of the laser beam spot was calculated as approximately ~14 MW/cm^2^.

The accelerating voltage between the sample holder and the grounded electrode is 17 kV. Ions are detected by the two channel plate detector (Hamamatsu, Photonics Deutschland GmbH, Herrsching am Ammersee, Germany) operating at the voltage of 2.3 kV. The signal from the detector is then sent to a 500 MHz (1 G sample/s) HP 54615B digital oscilloscope (Hewlett Packard, Warsaw, Poland) where all the data is collected as mass spectra. To enhance signal to noise ratio, each mass spectrum is averaged from 256 results obtained for consecutive laser shots. Next, the mass spectra are transferred to a PC for processing.

### Materials and Methods

For studies of the matrix effect on the MALDI TOF MS measurement synthetic PR-39 (RRRPRPPYLP RPRPPPFFPP RLPPRIPPGF PPRFPPRFP) with purity > 95 % as confirmed by high-performance liquid chromatography and mass spectrometry (NOVAZYM POLAND s.c. Poznan Science &Technology Park, Poznan) was used. The matrices: α-cyano-4-hydroxycinnamic acid (CCA), 2,5-dyhydroxybenzoic acid (DHB), sinapinic acid (SA), nicotinic acid, benzoic acid, and succinic acid were purchased from Sigma-Aldrich.

**Fig. 3:**
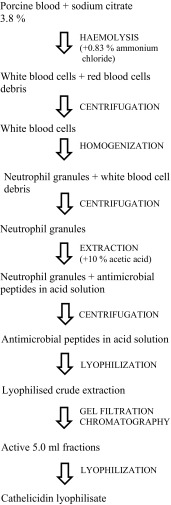
Successive steps of obtaining the cathelicidin liophylisate sample from the porcine blood

For the second part of investigations, all the cathelicidins (PR-39, PF-2, PG-1, PG-2, PG-3) were obtained from the porcine neutrophils crude extract in the process of crude extraction and gel filtration chromatography. Described method is dedicated for isolation of cationic antimicrobial peptides of low molecular mass. Successive steps of formation of a portion of cathelicidin lyophilisate, which was directly used for the MALDI TOF MS measurement, are shown in Fig. 3. Fresh porcine blood was collected at an abattoir using 3.8 % citrate as an anticoagulant. A crude extraction was obtained from blood neutrophils according to the method described previously [61]. Briefly, after lysis of red blood cells by the addition of 0.83 % ammonium chloride, white blood cells (with purity of 75–85 % of neutrophils) were collected by centrifugation at 700×*g*, 15 min, 4 °C. Then, the obtained cells were resuspended in the modified phosphate buffer saline (PBSX) buffer (137 mM NaCl, 2.7 mM KCl, 0.5 mM MgCl_2_, 8.1 mM Na_2_HPO_4_, 1.5 mM KH_2_ PO_4_, pH 7.4), and the cells were homogenized with DIAX 900 Heidolph (12.5 rpm, 15 min) to release the neutrophil granules. These granules were collected (25,000×*g*, 40 min, 4 °C), suspended in 10 % acetic acid and stirred overnight at 4 °C to extract the antimicrobial peptides. The solution containing the peptides was separated from the granules (25,000×*g*, 20 min, 4 °C) lyophilised and stored at −20 °C. Gel filtration chromatography was used to separate the components present in the crude extraction according to their sizes. The extract was passed through a Sephadex G-50 (Fine, Sigma-Aldrich) column, using a running buffer of 5 % acetic acid at 0.5 ml/min. The absorbance of the eluate (every 0.5 ml) was monitored at 280 nm. The 5.0-ml fractions were pooled and lyophilised [61].

The amount of a sample in the portion of lyophilisate was about 20 μg. Matrix solutions were prepared by dissolving 0.01 g of the matrix in 1 ml of ACN (acetonitrile) and 0.1 % TFA acid (1:1, *v*: *v*). To prepare the sample solution, the portion of lyophilisate or the synthetic PR-39 (20 μg) were dissolved in 1 ml of eluent (60 % ACN, 0.1 % TFA, 49.9 % distilled water). The authors used the dried-droplet sample deposition method: the adequate volume of the sample solution and the matrix solution was put directly onto the surface of the stainless steel sample holder, and the solvents were evaporated.

## Results and Discussion

**Fig. 4:**
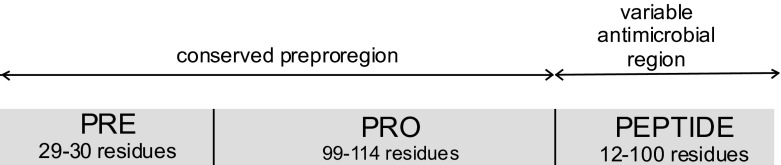
The schematic primary structure of cathelicidins

Cathelicidins are encoded in the genome as prepropeptides with a classical N-terminal signal peptide (conserved signal sequence), propiece (cathelin), and C terminal highly variable peptide [63, 64]. The schematic primary structure of cathelicidin is given in Fig. 4. Proteolytic processing of prepropeptide is necessary to release the mature antimicrobial peptide in its active form. After removal of a signal peptide by signal peptidase, cathelicidins are stored in neutrophil granules as inactive precursors (propeptides). The anionic prosegment functionally neutralizes the cationic peptide and maintains an inactive propeptide during intracellular transport and storage in neutrophil granules to avoid intracellular toxicity. For example, the human cathelicidin is found in specific granules of neutrophils in its 17 kDa (140 amino acid) form, cationic antimicrobial peptide (hCAP18) and during or after secretion may undergo processing to mature 5 kDa (37 amino acid) peptide (LL-37). Processing of cathelicidin proforms to mature antimicrobial peptides occurs by proteolytic cleavage of the prosequence upon degranulation of neutrophils activated by various stimuli. It is known that in cattle and pigs cleavage is mediated by elastase, whereas in humans by proteinase 3 [63].

### Choosing a Proper Matrix- Studies on Synthetic PR-39

**Fig. 5:**
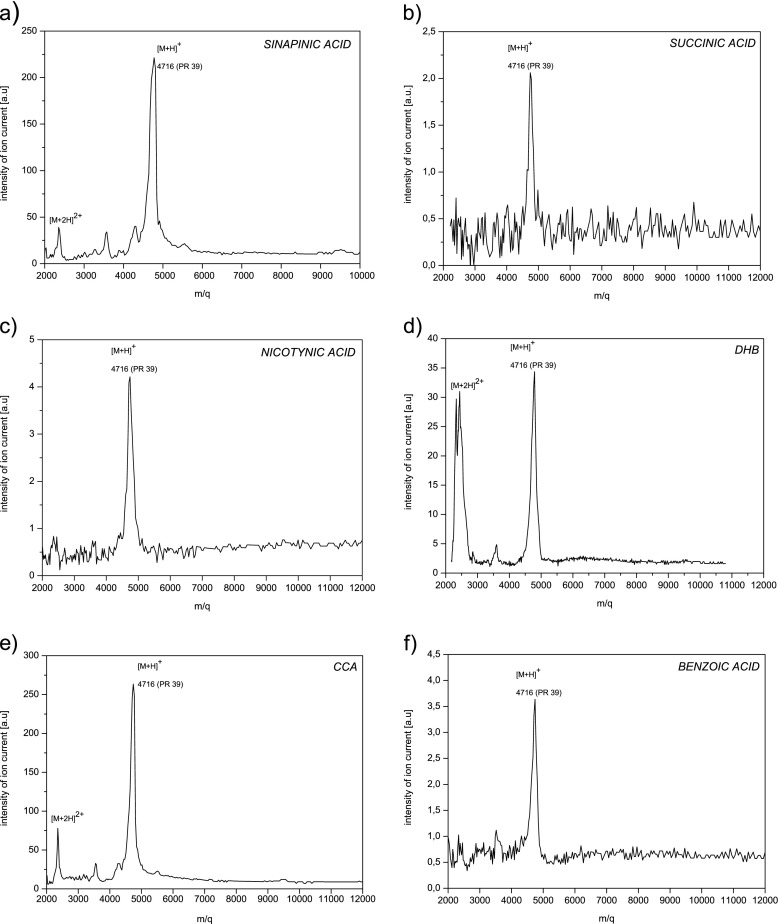
MALDI TOF mass spectra of the synthetic PR-39 (4716 Da) obtained for different matrices: **a**) sinapinic acid (SA), **b**) succinic acid, **c**) nicotinic acid, **d**) 2,5-dyhydroxybenzoic acid (DHB), **e**) α-cyano-4-hydroxycinnamic acid (CCA), **f**) benzoic acid

**Fig. 6:**
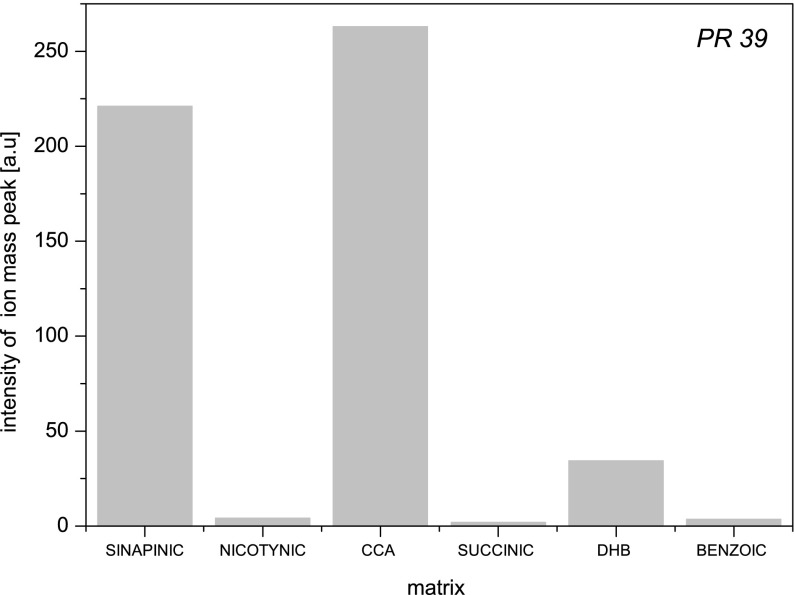
The intensity of the synthetic PR-39 ion mass peak as a function of the type of the matrix used in the sample preparation process

In Fig. 5, mass spectra of synthetic PR-39 obtained for different matrices are presented. In each of them, there is singly charged positive PR-39 ion mass peak visible ([M + H]^+^). The intensity of it depends dramatically on the matrix used in the measurement. It is the biggest for CCA and about 100 times smaller in case of succinic acid (Fig. 6). Only using of some matrices (CCA, DHB, sinapinic acid) leads to creation of double charged positive PR-39 ion mass peak ([M + 2H]^2+^). The intensity of it is significantly smaller comparing to molecular ion mass peak with the exception of DHB.

### Importance of the Matrix/Sample Ratio

**Fig. 7:**
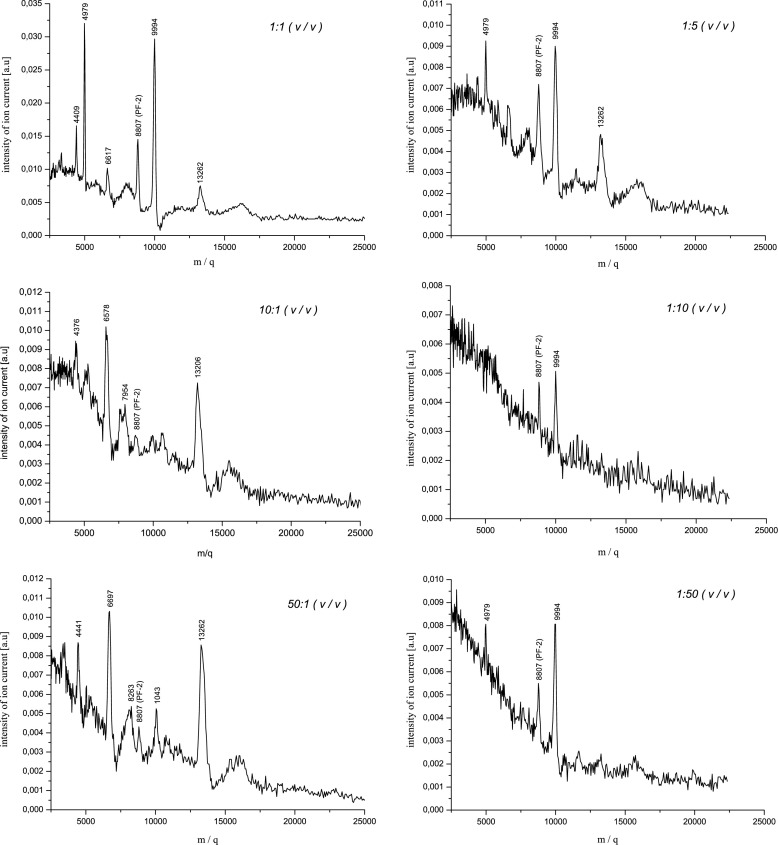
Mass spectra of the lyophilisate sample with the PF-2 (8807 Da) ion mass peak obtained for different matrix/sample ratios (*v*/*v*): 1:1 (**a**),1:5 (**b**), 10:1 (**c**), 1:10 (**d**), 50:1 (**e**), 1:50 (**f**)

Studies on the impact of the matrix/sample ratio on the mass spectra obtained were carried out by using CCA as a matrix, which appeared to be the optimal for MALDI TOF MS measurement of investigated cathelicidins. In Fig. 7, there can be seen mass spectra of the lyophilisate sample with PF-2 (8807 Da) ion mass peak obtained for different ratios of a matrix and a sample solution (*v*/*v*) in the measured mixtures of both solutions: 1:50, 1:10, 1:5, 1:1, 10:1, 50:1. All the tested ratios are summarized in Table 1. The peptide of 9994 Da reflects a cathelin-like fragment, whereas the ion mass peak of 13,262 Da corresponds to proprotegrin (prosegment consisted of cathelin-like prosequence and C-terminal domain before enzymatic cleavage — see Fig. 4) [65]. The fragments of 4979 and 6617 Da are the products of cleavage of porcine cathelicidins. The molecules of masses ranging from about 10 to 18 kDa represent various members of the cathelicidin family that contain the cathelin domain.

**Table 1:** All the matrix/sample ratios tested in the MALDI TOF MS studies of investigated cathelicidins contained in the lyophilisate sample

Matrix	Sample
1	100
1	50
1	10
1	2
1	1
2	1
10	1
50	1
100	1

**Fig. 8:**
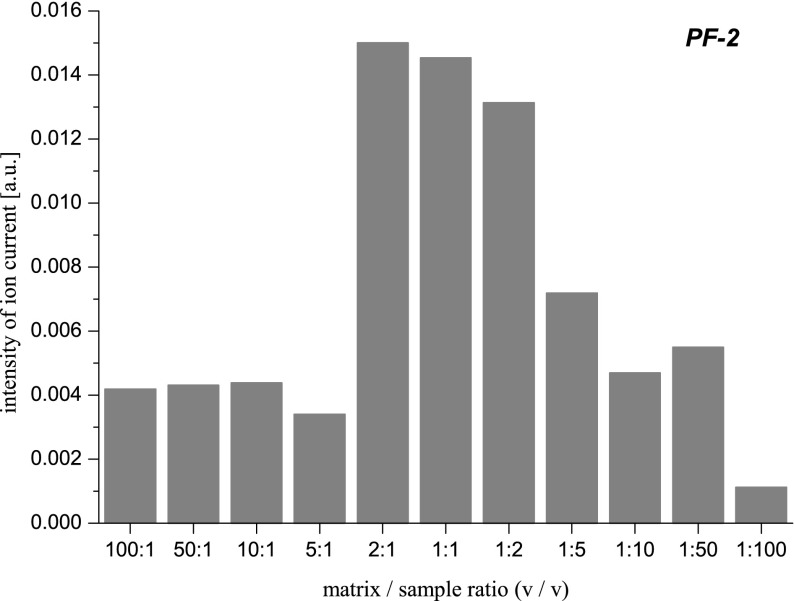
The intensity of the PF-2 ion mass peak as a function of the matrix/sample ratio used in the sample preparation process

As it can be seen, the intensity of individual ion mass peaks strongly depends on the sample preparation in the MALDI TOF MS measurement. Quantitatively, it is shown in Fig. 8. In the case of PF-2 ion mass peak, the sensitivity of the MALDI method is the highest for 2:1 matrix/sample ratio (*v*/*v*). Using other matrix/sample ratios, PF-2 peptide is also detected but the mass spectra obtained are much less clear. Depending on a way of the sample preparation, many other ion mass peaks also appear in the mass spectrum. For example, measuring with the 1:50 matrix/sample ratio 4979, 8807 and 9994 Da masses are detected while using the 50:1 matrix/sample ratio there are registered the masses: 4441, 6697, 8263, 8807, 1043, 13,262 Da. It is typical of the MALDI ion source where the quantity and quality of the produced sample ions is determined by the number of matrix ions created, which must be sufficient to ionize a number of specific molecules of the studied substance. The ions of these molecules are formed during their collisions with matrix ions. If the number of matrix ions is small, then adequately, only a small part of biomolecules namely those with higher affinities for charge than others are ionized. This can be seen as a small number of ion mass peaks in the mass spectrum and low intensity of ion current corresponding to the ionized biomolecules (see Fig. 7f). On the other hand, having too many matrix ions can lead to production of numerous kinds of different biomolecules ions, especially when the sample is complex, of natural origin, not cleaned and not mass separated before the MALDI TOF MS measurement. This, in turn, manifests itself as an illegible mass spectrum that can sometimes be even difficult to interpret (see Fig. 7e) or in the form of total ion mass suppression.

**Fig. 9:**
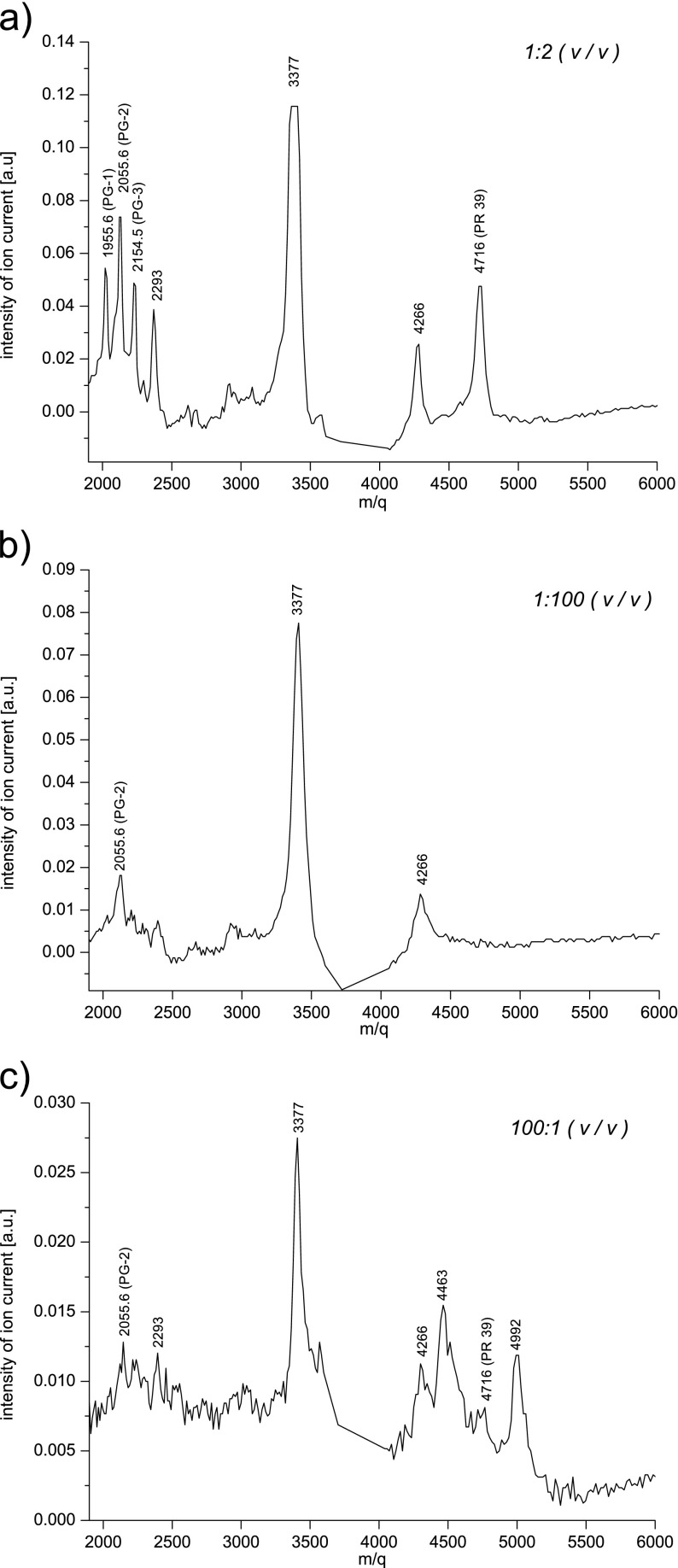
The mass spectra of the lyophilisate sample containing PG-1 (1955, 6 Da), PG-2 (2055, 6 Da), PG-3 (2154, 5 Da), and PR-39 (4716 Da) ion mass peaks obtained for the matrix/sample ratios (*v*/*v*): 1:2 (**a**), 1:100 (**b**), 100:1 (**c**)

**Fig. 10:**
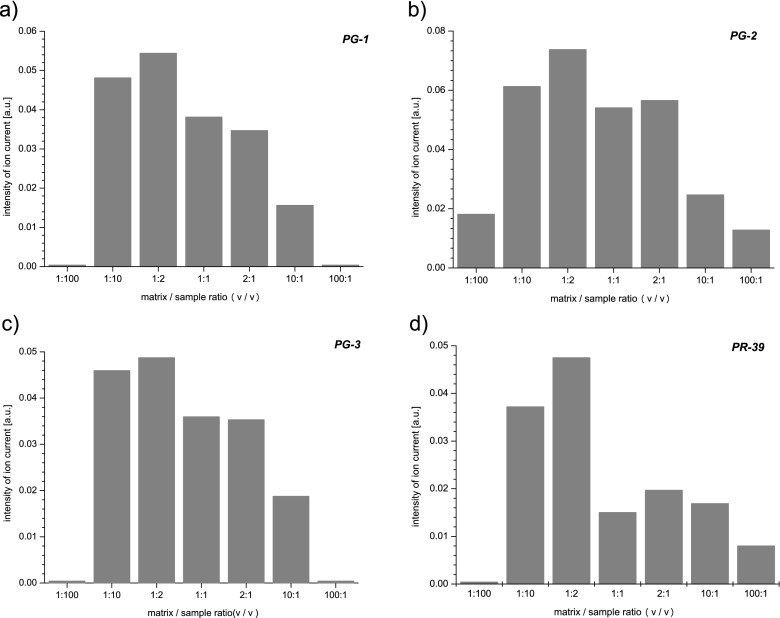
The intensity of the: PG-1 (**a**), PG-2 (**b**), PG-3 (**c**), and PR-39 (**d**) ion mass peak as a function of the matrix/sample ratio used in the sample preparation process

Analogous measurements to those for the PF-2 peptide were performed for cathelicidins PR-39 (4716 Da), PG-1 (1955,6 Da), PG-2 (2055,6 Da), and PG-3 (2154,5 Da). The mass spectra containing their ion mass peaks obtained for the matrix/sample ratios (*v*/*v*) 1:2, 1:100, and 100:1 are presented in Fig. 9. The mass spectrum obtained under optimal conditions of the sample preparation from the detection of studied cathelicidins point of view can be seen in Fig. 9a. Besides the ion mass peaks corresponding to the cathelicidins in this mass spectrum, there are also observed masses of 2293, 3377, and 4266 Da which come from proforms of cathelicidins created during the extraction process. Using all collected mass spectra, there were charts made (Fig. 10) showing the influence of the matrix/sample ratio on the intensity of ion mass peaks of particular cathelicidins. The chart clearly shows that for the investigated cathelicidins in the mass range 2000–6000 Da, the optimal matrix/sample proportion (v/*v*) is 1:2. Then, all studied cathelicidins are registered, and the intensity of ion mass peaks corresponding to them is the highest. Comparing mass spectra taken with extreme 1:100 and 100:1 matrix/sample proportions (*v*/*v*), there can be also seen big differences between them. As in the previous case (Fig. 7e and 7f), one can see that using an inappropriate amount of a matrix in the sample preparation process leads to detecting not all studied compounds (Fig. 9b) or registering too many ion mass peaks of different species present in a lyophilisate sample (Fig. 9c).

## Conclusions

Nowadays, MALDI TOF MS became an indispensable tool in qualitative, and in recent years also, more and more quantitative research of large biomolecules such as peptides, proteins, oligosaccharides, lipids, and many others contained in complex biological samples. Its main advantages are as follows: softness due to a matrix used in the sample preparation process, high sensitivity and high tolerance of impurities, and non-volatile buffers present in the sample. Additionally, through combination with a time of flight mass spectrometer (TOF MS), time of analysis is short, range of the measured masses is very high, and a mass spectrum obtained is simple to analyze compared to other methods of detection (for example electrospray mass spectrometry).

Despite its many advantages, a researcher must keep in mind that obtaining a good result in the MALDI TOF MS measurement depends largely on an appropriate sample preparation especially when the sample is not prior purified or is not earlier mass separated using chromatography methods. Mass analysis of complex biological samples such as lyophilisate from porcine neutrophils additionally requires careful optimization of the experimental conditions. Taking this issue into account, the authors took up the problem of the influence of the type and the amount of a matrix used for the measurement on the quality of a mass spectrum obtained. The results allow to draw several conclusions. The exact mass identification of all compounds present in the sample should be based on a clear mass spectrum with little noise to signal ratio and intensive ion mass peaks of the investigated analytes as far as the experimental conditions allow. The number and species of recorded ion mass peaks are closely correlated with the matrix/sample ratio (*v*/*v*) that was used in the sample preparation process. This inter alia attributed to the fact that in a mixture some analytes have always higher affinities for charge than others. To ionize a certain number of studied molecules, a sufficient number of matrix ions are required. In extreme cases, when the amount of a matrix relative to the sample is very high or very little, the analyte ions can be either suppressed or might not occur at all. However, there is a range of matrix/sample ratio in which the mass spectrum is measurable and contains all the investigated ion mass peaks. From the viewpoint of sensitivity of detection of studied biomolecules, the optimal matrix/sample ratio is different for particular compounds included in the sample and should be determined individually.
